# Genetic loci on chromosome 5 are associated with circulating levels of interleukin-5 and eosinophil count in a European population with high risk for cardiovascular disease

**DOI:** 10.1016/j.cyto.2016.01.007

**Published:** 2016-05

**Authors:** Olga McLeod, Angela Silveira, Elsa Valdes-Marquez, Harry Björkbacka, Peter Almgren, Karl Gertow, Jesper R. Gådin, Alexandra Bäcklund, Bengt Sennblad, Damiano Baldassarre, Fabrizio Veglia, Steve E. Humphries, Elena Tremoli, Ulf de Faire, Jan Nilsson, Olle Melander, Jemma C. Hopewell, Robert Clarke, Hanna M. Björck, Anders Hamsten, John Öhrvik, Rona J. Strawbridge, On behalf of the IMPROVE study group

**Affiliations:** a Cardiovascular Medicine Unit, Department of Medicine Solna, Karolinska Institutet, Stockholm, Sweden; b CTSU – Nuffield Department of Population Health, University of Oxford, Oxford, UK; c Experimental Cardiovascular Research Unit, Department of Clinical Sciences Malmö, Lund University, Malmö, Sweden; d Department of Clinical Sciences Malmö, Lund University, Malmö, Sweden; e Cardiovascular Medicine Unit, Department of Medicine, Science for Life Laboratory, Karolinska Institutet, Stockholm, Sweden; f Dipartimento di Scienze Farmacologiche e Biomolecolari, Università di Milano, Italy; g Centro Cardiologico Monzino, IRCCS, Milan, Italy; h Centre for Cardiovascular Genetics, University College London, UK; i Division of Cardiovascular Epidemiology, Institute of Environmental Medicine, Karolinska Institutet, Stockholm, Sweden; j Centre for Clinical Research Västerås, Uppsala University, SE-72189 Västerås, Sweden

**Keywords:** IMT_mean-max_, mean of the maximum IMT measures along the entire carotid tree, IMT-CC_mean_, mean of IMT measures in the common carotid, IMT-CC_max_, max of IMT measures in the common carotid, IMT-Bif_mean_, mean of IMT measures in the carotid bifurcation, IMT-Bif_max_, max of IMT measures in the carotid bifurcation, Subclinical atherosclerosis, Intima-media thickness, Genetics, IL-5, Eosinophil count, *RAD50*

## Abstract

IL-5 is a Th2 cytokine which activates eosinophils and is suggested to have an atheroprotective role. Genetic variants in the *IL5* locus have been associated with increased risk of CAD and ischemic stroke. In this study we aimed to identify genetic variants associated with IL-5 concentrations and apply a Mendelian randomisation approach to assess IL-5 levels for causal effect on intima-media thickness in a European population at high risk of coronary artery disease. We analysed SNPs within robustly associated candidate loci for immune, inflammatory, metabolic and cardiovascular traits. We identified 2 genetic loci for IL-5 levels (chromosome 5, rs56183820, BETA = 0.11, *P* = 6.73E^−5^ and chromosome 14, rs4902762, BETA = 0.12, *P* = 5.76E^−6^) and one for eosinophil count (rs72797327, BETA = −0.10, *P* = 1.41E^−6^). Both chromosome 5 loci were in the vicinity of the *IL5* gene, however the association with IL-5 levels failed to replicate in a meta-analysis of 2 independent cohorts (rs56183820, BETA = 0.04, *P* = 0.2763, *I*^2^ = 24, *I*^2^ − *P* = 0.2516). No significant associations were observed between SNPs associated with IL-5 levels or eosinophil count and IMT measures. Expression quantitative trait analyses indicate effects of the IL-5 and eosinophil-associated SNPs on *RAD50* mRNA expression levels (rs12652920 (r2 = 0.93 with rs56183820) BETA = −0.10, *P* = 8.64E^−6^ and rs11739623 (r2 = 0.96 with rs72797327) BETA = −0.23, *P* = 1.74E^−29^, respectively). Our data do not support a role for IL-5 levels and eosinophil count in intima-media thickness, however SNPs associated with IL-5 and eosinophils might influence stability of the atherosclerotic plaque via modulation of *RAD50* levels.

## Highlights

• Two loci identified for IL-5 levels on chromosomes and 14.
• One locus on chromosome 5 was identified for eosinophil count.
• The chromosome 5 loci are in close proximity, but independent and non-overlapping.
• IL-5-associated SNPs were not associated with carotid intima-media thickness.
• Eosinophil-associated SNPs were not associated with carotid intima-media thickness.

## 1 Introduction

Atherosclerosis is a process leading to coronary artery disease (CAD) and ischemic stroke, and is among the leading causes of mortality and morbidity throughout the world [1]. It is thought that a key component of atherosclerosis is lipid accumulation in the arterial wall, leading to leukocyte recruitment, inflammation and participation of both innate and adaptive immune responses. To support the immune responses hypothesis, a number of immune or inflammatory biomarkers for cardiovascular disease (CVD) have been investigated. Some, such as C-reactive protein (CRP), appear to reflect general disease processes [2], [3], whereas a number of cytokines (such as IL-1, IL-2, IL-6, IL-8, IL-10, IL-12, IL-17 and TNFα) are robustly associated with CVD and are thought to be actively involved in the inflammatory processes leading to atherosclerosis [4], [5].

Genetic studies have identified single nucleotide polymorphisms (SNPs) that are associated with increased risk of CAD in the interleukin-5 gene (*IL5*) [6] and increased risk of ischemic stroke in the interleukin-5 receptor alpha subunit gene (*IL5RA*) [7]. As with most genetic variants associated with CVD, the mechanism by which the *IL5* SNP influences disease is unclear. A number of studies (including one from our group) have also proposed an athero-protective role for IL-5 [8], [9], [10], [11], which is supported by the fact that it is produced mainly by anti-inflammatory T helper 2 (Th2) cells.

In addition to activated Th2 cells, IL-5 can be produced by mast cells and to a lesser extent by eosinophils. IL-5 is a growth and differentiation factor for B-cells, an immunoglobulin-A (IgA)-enhancing factor and a key cytokine in eosinophil maturation, differentiation, activation and survival [12], [13]; thus, it is interesting that the *IL5* locus has also been associated with eosinophil count [14]. Cellular responses are dependent upon IL-5 activating a dimeric receptor consisting of an α and a β-subunit. The β-subunit is shared with IL-3 and granulocyte–macrophage colony-stimulating factor (GM-CSF) [15], however the α-subunit is specific to IL-5. Thus, availability of the α-chain on the cell surface controls responsiveness of the cell to IL-5 [16], [17].

Thickness of the intima-media layer of the carotid artery can be considered a marker of cardiovascular risk. Differences in carotid intima-media thickness (IMT) over time can be indicative of changes in the structure, and possibly functions, of the vessel wall. The aims of this study were to identify genetic variants associated with IL-5 levels and to use these in a Mendelian randomisation approach to assess IL-5 levels for causal effects on carotid IMT. As eosinophil count and IL-5 levels are inter-related, we also investigated the genetic regulation of eosinophil count. We used the prospective IMPROVE study (the Carotid Intima Media Thickness [IMT] and IMT-Progression as Predictors of Vascular Events in a High Risk European Population study), which is based on 3703 subjects at high risk of CVD with extensive genetic data, biochemical phenotyping and repeat detailed ultra-sound measurements of the carotid artery IMT [18], [19]. Genetic markers robustly associated with increased levels of a biomarker or constituting a causal component of increased IMT should be useful for risk stratification and early prevention of CVD.

## 2 Materials and methods

### 2.1 Discovery cohort: the IMPROVE cohort

The design features, biobank and dataset of the IMPROVE study have been reported [18], [19]. Of 3703 participants with at least three cardiovascular risk factors but without symptoms or diagnosed prevalent disease, 3435 passed phenotypic quality control and were considered for the current study. Ethics committee approval for the study was obtained from each centre and written informed consent was obtained from all participants.

### 2.2 Phenotypes

A wide range of anthropometric and biochemical measurements were acquired, including fasting concentrations of HDL and LDL cholesterol (by Friedewald’s formula), triacylglycerol (TG), high sensitivity CRP (hs-CRP), creatinine and plasma glucose as described [19]. Diabetes was defined as diagnosis of diabetes and/or fasting blood glucose ⩾7 mmol/L and/or glucose lowering treatment. Levels of IL-5 were measured in EDTA-plasma samples using the ultra-sensitive kit for human IL-5 (MesoScale Discovery, Gaithersburg, MD, USA) following instructions provided by the manufacturer. Details of the high-resolution ultrasound measurements of carotid IMT at baseline and follow-up have been reported [18], [19]. A number of studies suggested that atherosclerotic changes can be site-specific and have distinct clinical implications [20], [21], [22]. Thus one composite and four segment-specific measurements of carotid IMT by were included in the study: IMT_mean-max_ (mean of the maximum IMT measures along the entire carotid tree), IMT-CC_mean_ and IMT-CC_max_ (mean and maximum of common carotid segment), IMT-BIF_mean_ and IMT-BIF_max_ (mean and maximum of the bifurcation). The ultrasonographic measurements were obtained at baseline and after 15 and 30 months. These three time points were used in linear regression to assess change over time in IMT [18], referred to here as progression of IMT. Characteristics of the IMPROVE cohort with available genetic and phenotypic data (*n* = 3435) are presented in Table 1.

### 2.3 Genetic analysis

Genotyping was performed using Illumina 200 K Cardio-Metabochip [23] and Immunochip [24] genotyping arrays (hence referred to as the combined chip). Whilst the combined chips do not have genome-wide coverage, they do have good coverage of candidate or robustly associated loci for immune, inflammatory, metabolic and cardiovascular traits, which allows for a less conservative threshold for significance compared to genome-wide platforms. Genotyping and calling were performed at the SNP Technology Platform (Uppsala University, Sweden Uppsala). Standard genetic quality control procedures were followed with exclusions for SNPs failing call rate (<95%) and Hardy–Weinberg (*p* < 5E^−7^) checks or with minor allele frequency (MAF) <1% and for subjects with cryptic relatedness or sex check failures. After quality control, 3433 individuals with genetic and phenotypic data were included in the analysis.

### 2.4 Replication cohorts: the PROCARDIS and MDC cohorts

Replication was sought in the PROCARDIS [25] and MDC cohorts [26], [27].

The PROCARDIS cohort consists of cases and controls from the UK, Sweden, Italy and Germany. All cases were diagnosed with CAD before the age of 66 years and most (80%) had a sibling with CAD before 66 years. Controls were recruited from the same populations and did not have a diagnosis of CAD, or a sibling with diagnosis of CAD, before 66 years of age. Plasma levels of IL-5 were measured in duplicate on the same plate by a sandwich immunoassay with electrochemiluminescence detection using a MesoScale Diagnostics platform (Gaithersburg, Maryland, USA). Genomewide genotyping of PROCARDIS was conducting using the Illumina 1 M or 610 K platforms. Genotype calling was performed at the Centre National du Genotypage, Paris or the Uppsala SNP Genotyping platform, Sweden. Standard quality control was conducted as detailed above.

The Malmö Diet and Cancer (MDC) study is a prospective population-based cohort study including 28,449 subjects recruited during 1991–1996. Subjects aged 45–69 years, living in Malmö, Sweden were eligible for participation. Between November 1991 and February 1994, every second enrolled subject was also invited to take part in a substudy of the epidemiology of carotid artery disease. This MDC-CardioVascular cohort consists of 6103 subjects (60% women), 5540 of whom also agreed to have blood collected under standardized fasting conditions. MDC was genotyped using the Illumina OmniExpressExome chip and standard quality control was conducted as detailed above. The methodology used for measuring IL-5 levels has previously been described [28]. Briefly, levels of cytokines released by mononuclear cells after stimulation (with CD3/CD28 beads) were analysed using a multiplex cytokine immunoassay (MesoScale Discovery, Gaithersburg, MD, USA).

Whilst the genotyping platforms utilised by the discovery and replication cohorts are not the same, all 3 platforms are produced by the same company and based upon the same bead chip technology. In addition, the same quality control parameters are used for the genotype calling, as well as for the downstream analyses (as detailed above).

### 2.5 Statistical methods

Differences between two groups according to sex were assessed by Wilcoxon Mann–Whitney ranksum test for continuous variables and Fisher’s exact test for binary variables (Table 1). Since a sex difference in IL-5 levels has been observed [11], we adjusted for sex. Risk factors and biomarkers significantly associated with plasma levels of IL-5 (by Spearman rank correlation) were considered for inclusion in regression models. Variables with skewed distributions were log-transformed prior to regression analysis. In both the analyses of IL-5 and of eosinophil count, the basic model was adjusted for population structure (as assessed by the first 3 multi-dimensional scaling components), age and sex. For IL-5 analyses, the extended model also included LDL and hsCRP. For eosinophil count, the extended model included BMI, fasting proinsulin concentration and white blood count. Variables for inclusion in the extended model were determined by backward stepwise selection in a multiple regression model. Linear regression analysis was used to assess associations between SNP genotypes and plasma IL-5 concentrations or eosinophil count, assuming an additive genetic model. The threshold for replication was set at *p* < 1.00E^−5^, whereas array-wide significance was set at *p* < 1.92E^−7^ (based on the calculation of the number of uncorrelated SNPs on the combined Metabo-Immuno chips as previously described [29]). Linear regression assuming an additive genetic model was also used for analysis of IMT measures, with adjustment for age, sex and population structure. Further adjustment for established CVD risk markers included age, sex, population structure, BMI, SBP, HDL, TG, hsCRP, current smoking and type 2 diabetes.

Replication analyses were conducted in the same manner as the discovery analyses, but the models were amended for PROCARDIS, where country was used instead of MDS components and case-control status was included as a covariate. Statistical analyses were performed in STATA (Statacorp, Houston, TX, USA) except chip-wide genetic analyses which were conducted using PLINK [30]. Because of high correlation between IMT measurements of carotid segments [19], correction for testing multiple phenotypes was not applied.

### 2.6 Gene expression analysis

Differential gene expression analysis for SNPs robustly associated with plasma IL-5 concentrations was performed. Firstly, in the Advanced Study of Aortic Pathology (ASAP) [31], where aortic adventitia (*n* = 132), aortic media (*n* = 137) and heart (*n* = 127) tissue samples were analysed. In brief, tissue samples were collected from patients undergoing elective surgery for aortic aneurysm and/or aortic valve repair. RNA was extracted from biopsy material and hybridised to Affymetrix ST 1.0 exon arrays (Santa Clara, CA, USA). DNA was extracted from whole blood and genotyped on the Illumina 610 W-Quad Bead Array (San Diego, CA, USA). SNPs with call rate >95% were used for imputation, and imputed SNPs with quality scores of MACH <0.3 were excluded. The ASAP study was approved by the ethics committee at Karolinska Institutet. Associations between SNP genotypes and gene expression were assessed assuming an additive genetic model. Significance was set at *p* < 0.0056 (Bonferroni-correction for 3 SNPs in 3 tissues). Secondly the publicly available MuTHER gene expression data was also interrogated [32]. Of the IL-5 or eosinophil-associated SNPs identified in IMPROVE, 5 were available in the MuTHER dataset, thus *p* < 0.01 (Bonferroni-correction for 5 SNPs in one tissue) was set for the level of significance. Finally, the publically available rSNPBase [33] was searched effects of SNPs on gene expression. As the data in this resource is qualitative, no threshold for significance was employed.

## 3 Results

### 3.1 Cardiovascular, metabolic, inflammatory or immune-associated SNPs vs IL-5 levels or eosinophil count

**Supplementary Fig. 1:**
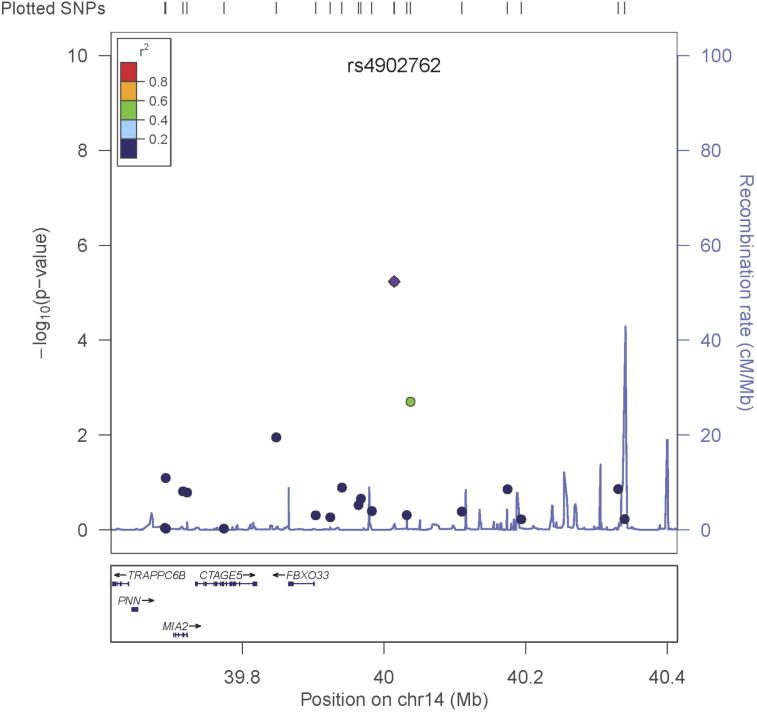
Regional plot for a locus on chromosome 14 associated with plasma IL-5 concentrations.

**Fig. 1:**
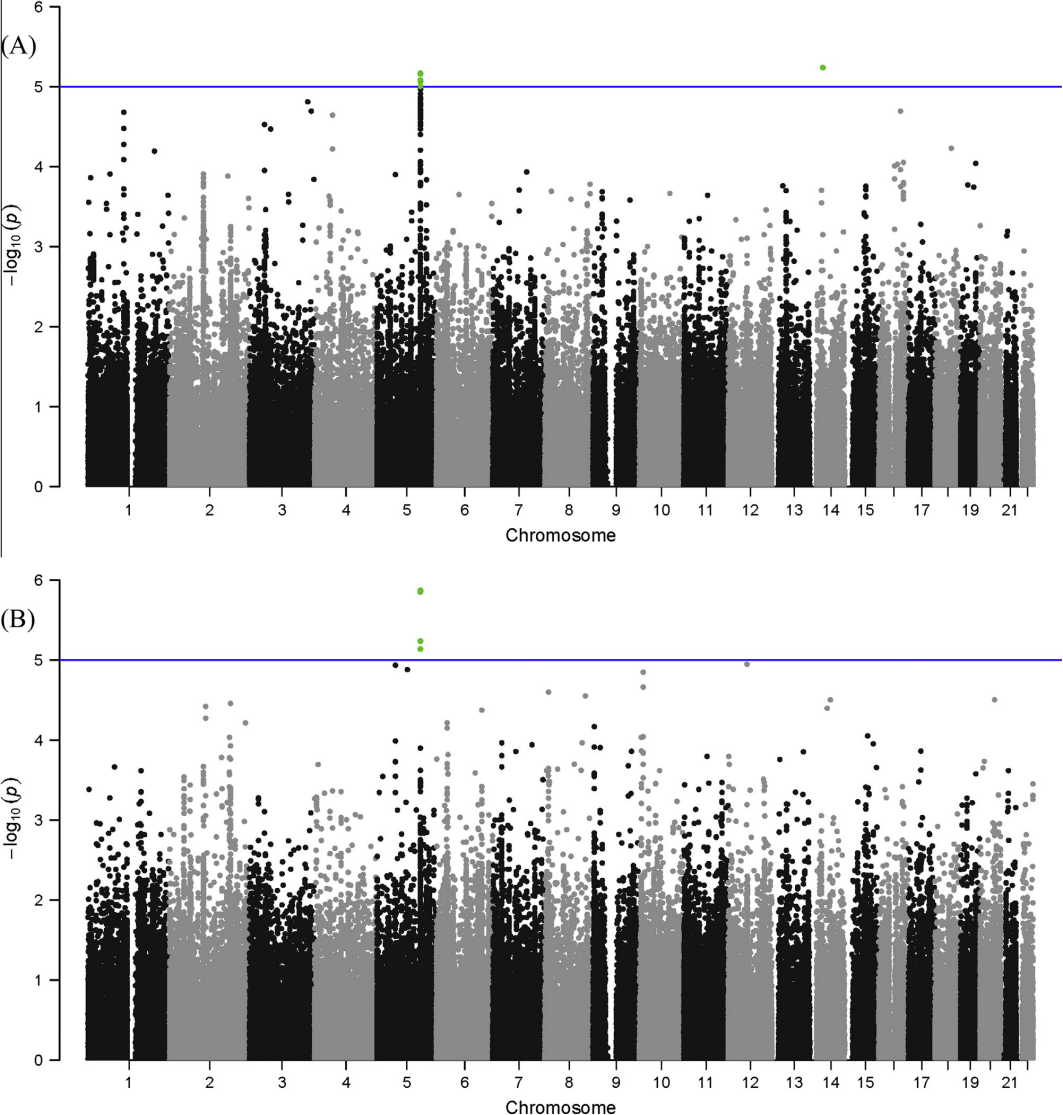
Manhattan plots of associations between SNPs on the combined chip with (A) plasma IL-5 levels and (B) eosinophil count. Associations were adjusted for age, sex and population stratification. The horizontal line at −log 10 *p* value = 5 indicates the threshold for replication.

In order to assess whether there are SNPs in loci previously associated with immune, inflammatory, metabolic or CVD traits which influence plasma levels of IL-5 or eosinophil count, the combined chip was analysed. When considering IL-5 levels, there were no genetic variants that demonstrated array-wide significant associations (i.e. *p* < 1.92E^−7^), however 2 loci reached the threshold set for replication (*p* < 1.00E^−5^, Fig. 1, Upper panel and Table 2). Further adjustment using the extended model had a negligible effect on the results (Table 2). The chromosome 14 locus consists of a single SNP (Supplementary Fig. 1), whilst the chromosome 5 locus (defined by the positions of the first and last SNPs reaching *p* < 1.00E^−5^ in the region) encompasses 79 SNPs extending across and upstream of the *IL5* gene. When considering associations with eosinophil count, one locus, including 14 SNPs, reached the threshold for replication but not array-wide significance (Fig. 1, Lower panel and Table 2) in both the basic and extended models (Table 2). This locus is also on chromosome 5 and extends across the *IL5* gene.

### 3.2 Genetic architecture of IL5 and eosinophil-associated loci

Given that only a single SNP was genotyped in the chromosome 14 locus, it is hard to say anything about the genetic architecture of this locus. More genotyping in this region would be needed to further explore this locus.

**Supplementary Fig. 2:**
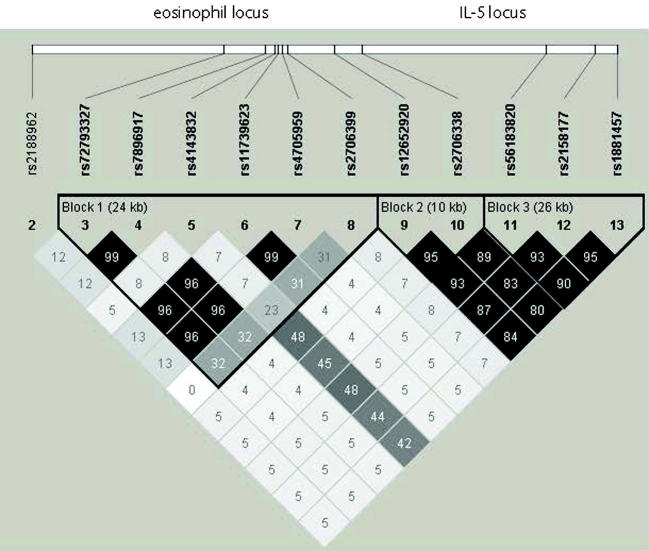
Linkage disequilibrium (LD) between candidate SNPs and those associated with IL5 levels or eosinophil count in the IMPROVE study (at *p* < 1.00E^−5^). Block 1 consists of eosinophil-associated SNPs and blocks 2 and 3 are IL5-associated SNPs. The candidate SNP rs2188962 (Irritable bowel disease-associated [34]) falls outside of the 2 novel loci described in this report, whilst the eosinophil count and CVD-associated SNPs (rs4143832 [14] and rs2706399 [6] respectively) are located within the novel eosinophil count-associated locus. Colours and values reflect R2 measures of LD.

**Fig. 2:**
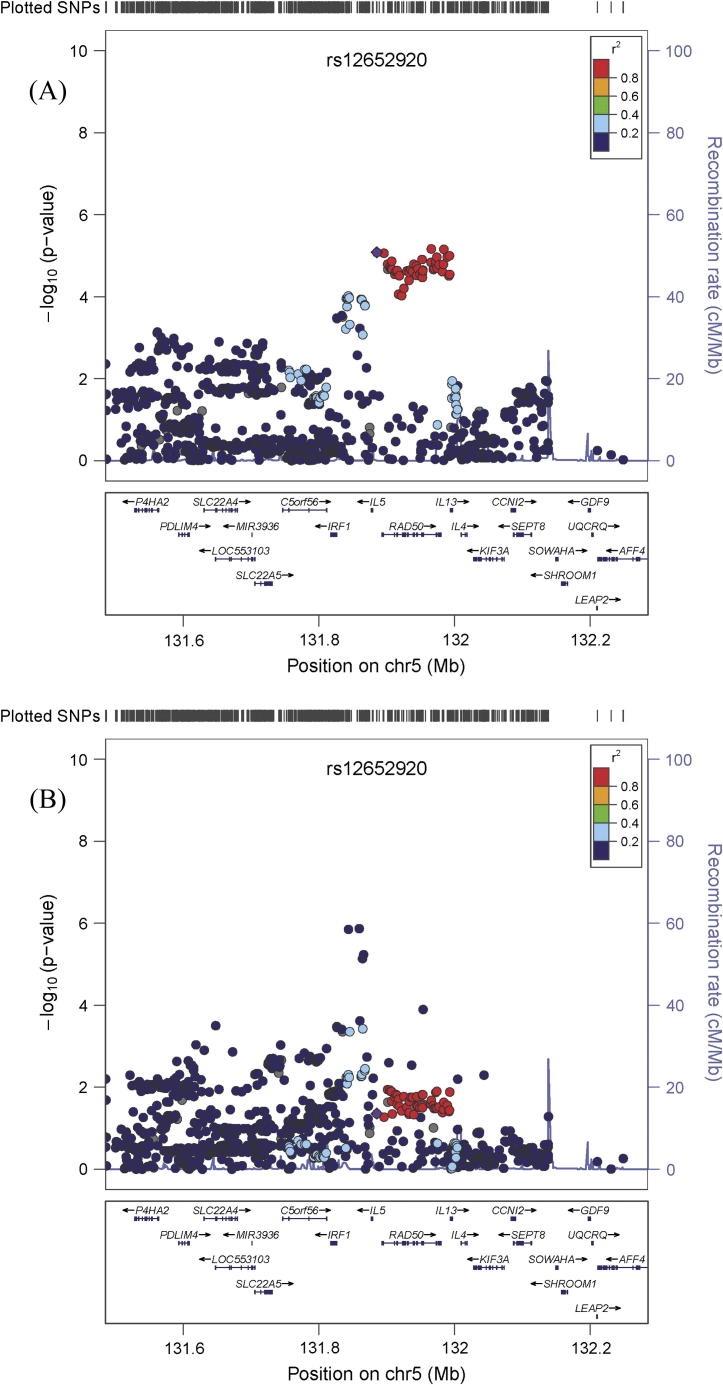
Regional plot of SNPs in the chromosome 5 loci which are associated with (A) circulating IL-5 concentrations and (B) eosinophil count. The purple diamond indicates rs12652920 (the lead SNP in the IL-5-associated locus), and thus demonstrates that the lead SNP in the eosinophil-associated locus (rs57896917) is independent by linkage disequilibrium.

The 2 loci on chromosome 5 discovered here are located in physical proximity to each other, however the SNPs in the 2 loci are distinct and appear to be independent from each other by linkage disequilibrium (LD, Fig. 2, Upper and Lower panels, Supplementary Fig. 2). The 3 genetic markers in the vicinity of the *IL5* gene reported in earlier studies were independent from each other and the loci identified in the current study as assessed by LD (Supplementary Fig. 2), however the eosinophil-associated SNP (rs4143832) and the CVD-associated SNP (rs2706399) fell within our eosinophil-associated locus. To further test the independence of the 2 loci, conditional analysis was conducted, whereby the IL-5 analysis was adjusted for the lead eosinophil-associated SNP and *vice versa*. As demonstrated in Table 3, there is negligible attenuation of the effect of the IL-5-associated SNPs on IL-5 levels when including the lead eosinophil-associated SNP as a covariate. The same is observed if the reverse is attempted, thus we conclude that the loci are independent.

### 3.3 Replication of IL5-associated loci

Ideally we would have liked to replicate both the IL-5 and eosinophil findings but due to lack of applicable cohorts it was only possible to attempt replication for the IL-5-assocaited SNPs. Characteristics of the replication cohorts are presented in Supplementary Table 1. Replication was attempted in 2 independent European cohorts for the IL-5-associated loci, namely rs56183820 on chromosome 5 and rs4902762 on chromosome 14. The size of effect in the replication meta-analysis (Table 4) was similar to that seen in the discovery analysis, however the direction was inconsistent and the associations were solidly non-significant.

### 3.4 IL-5 and eosinophil-associated SNPs in relation to IMT

Our aim was to use a Mendelian randomization approach to assess whether the IL-5 or eosinophil-associated loci influenced measures of IMT. As we were not able to replicate the association of the chr 5 locus with IL-5 levels, conducting a Mendelian randomization experiment was unjustified. However, we did assess the effect of these SNPs on IMT measures. Considering 6 independent loci (3 previously reported SNPs, 1 eosinophil-associated locus and 2 IL-5-associated loci), the Bonferroni-corrected significance threshold was set at *p* < 0.0083. No associations were observed between the SNPs and baseline measures of IMT (Supplementary Table 2). Eosinophil-associated SNPs demonstrated nominally significant associations with progression of IMT-CC_max_, but only rs2706399 (the G allele of which was previously associated with increased CVD risk [6]) met the Bonferonni-corrected p for significance (G allele, Beta −0.008, 95%CI (−0.012, −0.004), *p* = 0.0017, Supplementary Table 3). Further adjustment for emerging or established CVD risk markers had negligible effect on the associations (Supplementary Table 3). Inclusion of eosinophil count in the model rendered the eosinophil-associated SNPs non-significant, but did not have an effect on the association of rs2706399 (CVD-associated) with IMT-CC_max_ progression (Supplementary Table 3).

### 3.5 Expression QTL

To identify functional effects of the IL-5 or eosinophil-associated loci, SNPs which met the threshold for *p* < 1.00E^−5^ were examined for influence on expression of genes at the locus. In the ASAP study, aorta (adventitia and media) and heart tissues were examined. A region 250 kb up and downstream of the lead SNP was defined for each locus, and genes within this region were assessed for genotype-specific expression. Bonferroni correction was made for 3 tissues and 4 loci (one on chromosome 14, 3 on chromosome 5, *p* for significance <0.0042, (Supplementary Fig. 2)). The chromosome 14 region included 2 genes, *CTAGE5* and *FBXO33* and rs4902762 was associated with *CTAGE5* expression levels in heart tissue (*p* = 0.0073), but this did not reach statistical significance. The chromosome 5 eosinophil- and IL-5-associated loci overlapped to a large degree, and included many genes (*P4HA2* and *PDLIM4* (eosinophil count locus), *SLC22A4, SLC22A5, IRF1, IL5, RAD50, IL13, IL4, KIF3A* and *SEPT8* (both loci)*, SHROOM1, GDF9, UQCRQ, LEAP2* and *AFF4* (IL-5 locus). Rs12652920 was associated with *RAD50* expression in aortic media (*p* = 0.0093), but this did not reach the level of statistical significance. The MuTHER lymphocyte data [32] supports a positive effect of the rs4705959 T allele (associated with increased eosinophil count) on mRNA expression of *RAD50* and *SLC22A5*, but a negative effect on expression of *IL5* (Table 5). The C allele of rs12652920 (associated with increased IL-5 levels) was also associated with increased mRNA expression of *RAD50* (Table 5). The rSNPBase was queried to identify proxies (r2 > 0.8) for the lead SNPs and whether they have been reported to influence expression of genes. For the IL-5-associated locus (rs56183420) 63 proxies were identified, of which 47 reported eQTL effects on *RAD50* but only 24 for *IL5*. Of note, 35 of these SNPs were available in the IMPROVE cohort, all showed associations (*p* < 1.00E^−4^) with IL-5 levels, but not eosinophil count. For the eosinophil-associated locus (rs72797327) 4 proxies were identified, of which 2 each were reported for effects on *IL-5* and *RAD50* expression, but all 4 reported effects on *SEPT8* expression. Of these SNPS, all 3 that were available in IMPROVE showed strong (*p* < 1.00E^−5^) effects on eosinophil but not IL-5 levels. Considering the chromosome 14 locus, 23 proxies were identified with 5 influencing *CTAGE5* expression and 7 influencing *FBXO33* levels. Only one proxy was available in IMPROVE and was associated with IL-5 levels but not eosinophil count.

## 4 Discussion

This study identified genetic regulators of plasma IL-5 levels and eosinophil count, however the lack of replication of the genetic associations precludes the ability to formally assess the causality of IL-5 levels on IMT. To the best of our knowledge this is the largest study with measurement of IL-5 levels and the first to investigate genetic regulation of IL-5 levels.

The genetic loci associated with IL-5 levels and eosinophil count are in close proximity to each other and in the vicinity of the *IL5* gene on chromosome 5, but act independently. Earlier studies identified SNPs in the region of the *IL5* and *IL5a* genes as being associated with inflammatory bowel disease (rs2188962 [34]), eosinophil count (rs4143832 [14]) and coronary artery disease (rs2706399 [6]). These variants were present on the combined chip and thus were also analysed. No association was observed between rs2706399 and either IL-5 levels or eosinophil count (Supplementary Table 5). In contrast, rs2188962 demonstrated nominal associations and rs4143832 demonstrated convincing associations with IL-5 levels and eosinophil count (Supplementary Table 5). In agreement with Gudbjartsson et al., the minor allele of rs4143832 was associated with eosinophil count [14] as well as IL-5 levels. Of note, whilst these SNPs demonstrate some significance in relation to levels of IL-5 or eosinophil count, they do not reach the preset level of significance for being forwarded to replication analysis. Rs4143832 and rs2706399 are located within the eosinophil-associated locus reported here but are independent from the SNPs studied here (by LD, Supplementary Table 2).

Whilst SNPs at the loci identified here do show some associations with *IL5* mRNA levels, expression of the *RAD50* gene demonstrates stronger and more consistent associations across the loci. The results from rSNPBase are less clear, and whilst they do support *RAD50* as a possible candidate, they suggest that *SEPT8* (chromosome 5) and *FBXO33* (chromosome 14) might also be relevant. Recently, up-regulation of RAD50 and other double-strand repair proteins was reported in vascular smooth muscle cells from atherosclerotic plaque [35]. The authors suggest that these proteins are associated with plaque stability rather than atherosclerosis. Thus it is plausible that the *IL5* locus influences plaque stability via RAD50-associated mechanisms rather than IL-5-associated mechanisms. This speculative mechanism fits with the *IL5* locus being associated with MI [6] and unstable angina [36], typically resulting from plaque rupture, but not with IMT, which is indicative of vessel wall remodelling.

Eosinophil count and IL-5 levels are closely inter-connected: IL-5 is essential for eosinophil maturation and differentiation [37]. Conversely, eosinophil-specific granules store IL-5 [38], indicating that these cells might also contribute to increasing circulating IL-5 levels. In IMPROVE, there is a strong correlation between IL-5 concentrations and eosinophil count (Spearmans rho = 0.560, *p* < 0.001) [11]. Thus it is interesting that these 2 variables are regulated by independent loci in the same genetic region. One possibility is that a common factor, potentially RAD50, regulates both IL-5 levels and eosinophil count. An alternative scenario is that, given the roles of IL-5 and eosinophils in inflammation, upregulation of *RAD50* might be a DNA-protective stress response mechanism. This would explain the puzzling finding that the allele associated with increased eosinophil count shows increased *RAD50* and the inverse effect on *IL5* gene expression, whilst the allele associated with increased IL-5 levels also shows increased *RAD50* gene expression. It is of note that 2 of the top eQTL SNPs for *RAD50* [39] are those with the strongest associations with eosinophils (rs11739623 and rs4705959) in IMPROVE. Previous reports have demonstrated the use of studying specific immune cell types to explore immune or inflammation-related diseases [40], [41]. Whilst the relationship between the SNPs, *RAD50* expression, IL-5 levels and eosinophil counts requires clarification, these findings are of potential relevance for eosinophil-associated disorders.

Whilst this is the largest study with detailed IMT measures, plasma IL-5 concentrations and extensive genetic data for all subjects, there are limitations to the study. This study was conducted in subjects with at least 3 classical CVD risk factors, thus findings might not pertain to other populations. A sex difference in IL-5 levels has been reported [11], thus sex-stratified analyses would be of interest. However, this approach has not been used here, for two reasons: the distribution of outliers (⩾1.5 interquartile range above the upper quartile) between men and women might explain the sex difference in levels. Because of this and the loss of power when stratifying the analyses, we believe that the chance of detecting false positive signals is too high for this analysis to be robust. The lack of Th2 count or measurements of Th2 cytokines mean that we cannot conclusively determine the origin of IL-5 and whether Th2 responses are activated in these subjects. However, it has previously been shown that the percentage of Th2 cells has only a very weak (non significant) correlation with IL-5 levels released by activated leukocytes [28]. The genotyping chips employed in this study are based on a large scale candidate gene/loci experiment, which allows a less stringent significance threshold for the discovery analysis, however the whole genome is not represented. Thus we cannot comprehensibly describe the genetic architecture of the IL-5-associated locus on chromosome 14, nor can we exclude the possibility that true IL-5-influencing genetic variants exist which lie within areas of the genome not examined in this study.

## 5 Conclusions

This study identified genetic loci on chromosome 5 associated with IL-5 levels and eosinophil count. Whilst these loci are close together in the vicinity of the *IL5* gene, they are independent. Furthermore, expression analysis suggests *RAD50*, rather than *IL5*, as a prime candidate gene for these associations. An additional locus associated with IL-5 levels was observed on chromosome 14. This report suggests that these loci have negligible influence on IMT measures.

## Figures and Tables

**Table 1:** Clinical characteristics of the IMPROVE cohort.

	All subjects	Males	Females	*P*-value^a^
	3435	1662	1773	
Age (years)	64.5 (59.6–67.2)	64.3 (59.4–67.1)	64.7 (59.9–67.3)	0.030
Height (m)	1.67 (1.6–1.74)	1.74 (1.70–1.79)	1.60 (1.57–1.65)	<0.001
BMI (kg/m^2^)	26.8 (24.3–29.5)	27.1 (25.0–29.3)	26.4 (23.5–29.7)	<0.001
Waist/hip ratio	94 (86–102)	0.96 (0.93–1.01)	0.87 (0.82–0.91)	<0.001
SBP (mmHg)	140 (130–153)	141 (130–154)	140 (130–152)	0.260
DBP (mmHg)	81 (75–88)	82 (77–90)	80 (75–88)	<0.001
Hypertension (%)	2471 (72.0)	1168 (70.3)	1303 (73.5)	0.020
Total cholesterol (mmol/L)	5.44 (4.69–6.24)	5.20 (4.52–5.94)	5.67 (4.89–6.45)	<0.001
LDL-cholesterol (mmol/L)	3.5 (2.82–4.22)	3.37 (2.74–4.02)	3.65 (2.94–4.39)	<0.001
HDL-cholesterol (mmol/L)	1.2 (1.01–1.46)	1.10 (0.93–1.30)	1.32 (1.11–1.60)	<0.001
Triglycerides (mmol/L)	1.31 (0.94–1.9)	1.37 (0.97–2.02)	1.26 (0.91–1.79)	<0.001
Fasting glucose (mmol/L)	5.5 (5–6.3)	5.7 (5.2–6.6)	795.3 (4.8–6.0)	<0.001
Diabetes mellitus (%)	904 (26.8)	525 (32.2)	379 (21.7)	<0.001
CRP (mg/L)	1.86 (0.77–3.58)	1.63 (0.67–3.25)	2.10 (0.91–3.92)	<0.001
Current smokers (%)	515 (15.0)	276 (16.6)	239 (13.5)	0.006
Vitamin D (nmol/L)	48 (34–64)	50 (37–65)	47 (30–62)	<0.001
IL-5 (pg/mL)	0.43 (0.27–0.71)	0.48 (0.30–0.76)	0.39 (0.25–0.64)	<0.001
Eosinophils (%)	2.6 (1.7–4.0)	2.8 (1.8–4.1)	2.5 (1.6–3.8)	<0.001
Framingham risk score	0.23 (0.15–0.35)	0.32 (0.23–0.45)	0.16 (0.11–0.24)	<0.001
Lipid-lowering therapy (%)	1675 (49.6)	785 (48.2)	890 (50.9)	0.060
Anti-hypertensive therapy (%)	1936 (56.4)	897 (54.0)	1039 (58.6)	0.005

*Baseline IMT*				
IMT_mean_	0.85 (0.74–1)	0.90 (0.78–1.06)	0.80 (0.71–0.93)	<0.001
IMT_max_	1.85 (1.39–2.5)	2.03 (1.55–2.68)	1.74 (1.30–2.31)	<0.001
IMT_mean-max_	1.19 (1.04–1.41)	1.27 (1.09–1.51)	1.13 (0.99–1.31)	<0.001
IMT-CC_mean_	0.71 (0.65–0.8)	0.74 (0.66–0.83)	0.70 (0.64–0.77)	<0.001
IMT-CC_max_	1.07 (0.96–1.3)	1.13 (0.98–1.40)	1.03 (0.94–1.19)	<0.001
IMT-BIF_mean_	1.06 (0.85–1.34)	1.12 (0.91–1.45)	1.00 (0.80–1.24)	<0.001
IMT-BIF_max_	1.67 (1.3–2.22)	1.84 (1.39–2.41)	1.57 (1.26–2.13)	<0.001

*IMT progression*				
IMT_mean_	0.017 (−0.0001–0.036)	0.019 (−0.0002–0.039)	0.015 (0.0001–0.032)	0.004
IMT_max_	0.036 (−0.035–0.112)	0.036 (−0.036–0.116)	0.034 (−0.033–0.108)	0.110
IMT_mean-max_	0.021 (−0.005–0.051)	0.024 (−0.003–0.058)	0.019 (−0.005–0.047)	<0.001
IMT-CC_mean_	0.007 (−0.006–0.022)	0.009 (−0.006–0.025)	0.006 (−0.006–0.020)	0.006
IMT-CC_max_	0.007 (−0.020–0.042)	0.011 (−0.023–0.050)	0.004 (−0.019–0.039)	0.010
IMT-BIF_mean_	0.029 (−0.004–0.069)	0.031 (−0.005–0.073)	0.027 (−0.003–0.066)	0.230
IMT-BIF_max_	0.036 (−0.020–0.113)	0.037 (−0.028–0.114)	0.036 (−0.012–0.112)	0.590

Where: BMI, body mass index; SBP, systolic blood pressure; DBP, diastolic blood pressure; LDL, low-density lipoprotein; HDL, high-density lipoprotein; CRP, C-reactive protein. Hypertension: diagnosis of hypertension and/or treatment with antihypertensive drugs; Diabetes: diagnosis of diabetes and/or treatment with insulin or other hypoglycemic drug, and/or fasting glucose ⩾7 mmol/L at the baseline examination. Values are expressed as median (interquartile range) or number of subjects in group (%).

a *P*-values between two groups were calculated by Mann–Whitney U test for continuous variables, and by Fisher’s exact test for binary variables.

**Table 2:** Associations reaching the threshold for replication with IL5 levels or eosinophil count.

						IL-5 (*n* = 3433)						Eosinophils (*n* = 2365)					
						Basic model			Extended model			Basic model			Extended model		
Trait	SNP	CHR	MAF	MA	RA	BETA	SE	*P*	BETA	SE	*P*	BETA	SE	*P*	BETA	SE	*P*
Eonsinophil	rs72797327	5	0.249	G	A	−0.05	0.02	0.0249	−0.05	0.02	0.0427	−0.10	0.02	1.41E^−06^	−0.08	0.02	5.83E^−05^
rs57896917	5	0.250	T	A	−0.05	0.02	0.0255	−0.05	0.02	0.0438	−0.10	0.02	1.35E^−06^	−0.08	0.02	5.60E^−05^
rs11739623	5	0.244	T	C	−0.05	0.02	0.0413	−0.04	0.02	0.0670	−0.09	0.02	7.28E^−06^	−0.08	0.02	4.35E^−05^
rs4705959	5	0.244	C	T	−0.05	0.02	0.0349	−0.04	0.02	0.0565	−0.09	0.02	5.81E^−06^	−0.09	0.02	3.46E^−05^

IL5	rs12652920	5	0.221	C	G	0.11	0.02	8.20E^−06^	0.11	0.02	6.30E^−06^	0.05	0.02	0.0441	0.04	0.02	0.0579
rs2706338	5	0.215	T	C	0.11	0.02	8.55E^−06^	0.11	0.02	6.23E^−06^	0.05	0.02	0.0534	0.04	0.02	0.0692
rs56183820	5	0.220	T	C	0.11	0.02	6.73E^−06^	0.11	0.02	5.58E^−06^	0.05	0.02	0.0207	0.05	0.02	0.0264
rs2158177	5	0.213	G	A	0.11	0.02	6.87E^−06^	0.11	0.02	7.04E^−06^	0.05	0.02	0.0233	0.05	0.02	0.0300
rs1881457	5	0.215	C	A	0.11	0.02	9.86E^−06^	0.11	0.02	1.11E^−05^	0.05	0.02	0.0363	0.04	0.02	0.0496
rs4902762	14	0.156	A	G	0.12	0.03	5.76E^−06^	0.12	0.03	1.08E^−05^	0.01	0.03	0.6667	0.02	0.02	0.3916

Where: Trait, trait with which the locus is associated; MAF, minor allele frequency; MA, minor allele; RA, reference allele; basic model, adjustment for age, sex and population structure; extended, adjustment for age, sex, population structure and (for IL5 analysis) lipids and crp or (for eosinophil count analysis) white blood cell count and proinsulin.

**Table 3:** Association conditional on a top SNP from the neighbour loci.

						IL5 (*n* = 3433)						Eosinophils (*n* = 2365)					
						Basic			+Lead eosinophil-associated SNP (rs57896917)			Asmds			+Lead IL5-associated SNP (rs56183820)		
Trait	SNP	CHR	MAF	MA	RA	BETA	SE	*P*	BETA	SE	*P*	BETA	SE	*P*	BETA	SE	*P*
Eonsinophil	rs72797327^a^	5	0.249	G	A	−0.05	0.02	0.0249	na	na	na	−0.10	0.02	1.41E^−06^	−0.09	0.02	9.90E^−06^
rs57896917	5	0.250	T	A	−0.05	0.02	0.0255	na	na	na	−0.10	0.02	1.35E^−06^	−0.09	0.02	9.51E^−06^
rs11739623	5	0.244	T	C	−0.05	0.02	0.0413	0.14	0.11	0.1999	−0.09	0.02	7.28E^−06^	−0.09	0.02	4.60E^−05^
rs4705959	5	0.244	C	T	−0.05	0.02	0.0349	0.11	0.11	0.2989	−0.09	0.02	5.81E^−06^	−0.09	0.02	3.74E^−05^

IL5	rs12652920	5	0.221	C	G	0.11	0.02	8.20E^−06^	0.10	0.02	4.26E^−05^	0.05	0.02	0.0441	0.04	0.10	0.6932
rs2706338	5	0.215	T	C	0.11	0.02	8.55E^−06^	0.10	0.02	4.31E^−05^	0.05	0.02	0.0534	0.04	0.07	0.5655
rs56183820	5	0.220	T	C	0.11	0.02	6.73E^−06^	0.10	0.02	3.99E^−05^	0.05	0.02	0.0207	na	na	na
rs2158177	5	0.213	G	A	0.11	0.02	6.87E^−06^	0.10	0.02	3.85E^−05^	0.05	0.02	0.0233	0.05	0.09	0.5677
rs1881457	5	0.215	C	A	0.11	0.02	9.86E^−06^	0.10	0.02	5.51E^−05^	0.05	0.02	0.0363	0.04	0.08	0.6372

Where: Trait, trait with which the locus is associated; MAF, minor allele frequency; MA, minor allele; RA, reference allele; basic model, adjustment for age, sex and population structure; na, not applicable.

a rs72797327 is in almost complete LD (r2 = 0.99) with the SNP included as a covariate, thus results are not reported for this SNP.

**Table 4:** Replication meta-analysis of IL5-associated genetic variants.

					Basic model						Extended model					
SNP	CHR	MAF	MA	RA	BETA	SE	*P*	Direction	I2	I2_p	BETA	SE	*P*	Direction	I2	I2_p
rs56183820	5	0.21	T	C	0.04	0.04	0.2763	− +	23.9	0.2516	0.05	0.04	0.2517	− +	32.3	0.2242
rs4902762	14	0.14	A	G	-0.02	0.05	0.6568	− −	0	0.5668	−0.02	0.05	0.6111	− −	0	0.4814

Where: MAF, minor allele frequency; MA, minor allele; RA, reference allele; basic model, adjustment for age, sex and population structure; extended, adjustment for age, sex, population structure, lipids and crp; direction, direction of minor allele effect; I2, I squared measure of heterogeneity; I2_p, *P* for heterogeneity. Cohorts included in the meta-analysis are MDC and PROCARDIS.

**Table 5:** Effect of alleles associated with increased eosinophil counts or IL-5 levels on gene expression in the MuTHER lymphocyte dataset.

Trait	SNP	CHR	SNP_INFO	Gene	EA	EAF	BETA	SE	*P*
Eonsinophil	rs11739623	5	0.999	*RAD50*	T	0.252	−0.23	0.02	1.74E^−29^
*SLC22A5*	T	0.252	−0.05	0.01	4.75E^−04^
*IL5*	T	0.252	0.02	0.01	8.52E^−04^
*P4HA2*	T	0.252	0.07	0.02	0.0026

	rs4705959	5	0.996	*RAD50*	T	0.749	0.23	0.02	2.06E^−29^
	*SLC22A5*	T	0.749	0.05	0.01	2.95E^−04^
	*IL5*	T	0.749	−0.02	0.01	6.52E^−04^
	*P4HA2*	T	0.749	−0.08	0.02	0.0021

IL5	rs12652920	5	0.977	*RAD50*	G	0.808	−0.10	0.02	8.64E^−06^
*P4HA2*	G	0.808	0.07	0.03	0.0072

	rs2706338	5	0.986	*RAD50*	T	0.194	0.10	0.02	1.02E^−05^
	*P4HA2*	T	0.194	−0.07	0.03	0.0095

	rs4902762	14	0.95	*TRAPPC6B*	G	0.841	0.05	0.01	0.0013
	*PNN*	G	0.841	0.09	0.03	0.0066

Where: EA, Effect allele; EAF, effect allele frequency; The rs4705959 T allele was associated with increased eosinophil levels; The rs12652920 C allele was associated with increased IL-5 levels.
